# Study on the Antinociceptive Activity and Mechanism of Action of Isolated Saponins from *Siolmatra brasiliensis* (Cogn.) Baill

**DOI:** 10.3390/molecules24244584

**Published:** 2019-12-14

**Authors:** Thais Biondino Sardella Giorno, Carlos Henrique Corrêa dos Santos, Mario Geraldo de Carvalho, Virgínia Cláudia da Silva, Paulo Teixeira de Sousa, Patricia Dias Fernandes, Fabio Boylan

**Affiliations:** 1 Federal University of Rio de Janeiro, Institute of Biomedical Sciences, Laboratory of Pharmacology of Pain and Inflammation, Rio de Janeiro 21941-902, Brazil; 2 Federal University of Rio de Janeiro, Institute of Biomedical Sciences, Graduate Program in Pharmacology and Medicinal Chemistry, Rio de Janeiro 21941-902, Brazil; 3 Federal Rural University of Rio de Janeiro, Department of Chemistry, Seropédica 23890-000, Brazil; 4 Federal University of Mato Grosso, Department of Chemistry, Cuiabá 78935-901, Brazil; 5 Trinity College Dublin, Trinity Biomedical Sciences Institute, School of Pharmacy and Pharmaceutical Sciences, Dublin 2, Ireland

**Keywords:** *Siolmatra brasiliensis*, antinociceptive activity, cayaponoside A1, cayaponoside B4, cayaponoside D, siolmatroside I, pain, analgesia

## Abstract

Infusions of roots of *Siolmatra brasiliensis* (Cogn.) Baill, (“taiuiá”, “cipó-tauá”) are used for toothache pain and ulcers. We aimed to study the antinociceptive effects and identify the possible mechanism of action of this plant and its isolated substances (cayaponoside A1, cayaponoside B4, cayaponoside D, and siolmatroside I). Hydroethanol extract (HE), ethyl acetate fraction (EtOAc), and isolated saponins were evaluated in chemical and thermal models of pain in mice. Animals were orally pretreated and evaluated in the capsaicin- or glutamate-induced licking and in the hot plate tests. The antinociceptive mechanism of action was evaluated using the hot plate test with the following pretreatments: Atropine (cholinergic antagonist), naloxone (opioid antagonist), or L-NAME (nitric oxide synthase inhibitor). All extracts and isolated saponins increased the area under the curve in the hot plate test. Tested substances induced a higher effect than the morphine-treated group. Our data suggest that stems of *S. brasiliensis* and their isolated substances present antinociceptive effects. Cholinergic and opioidergic pathways seem to be involved in their mechanism of action. Taken together our data corroborate the traditional use of the plant and expands the information regarding its use.

## 1. Introduction

*Siolmatra brasiliensis* (Cogn.) Baill is a climbing plant belonging to the Cucurbitaceae family that occurs in the central region of Brazil, especially in Cerrado and Pantanal where it is popularly known as “taiuiá” or “cipó-tauá” [1]. Its roots are considered a purifying and antisyphilis agent [2]. Infusions prepared with roots are widely used in traditional medicine as an analgesic for treatment of toothache [2] and for the treatment of ulcers [3]. Due to the presence of cucurbitacins, compounds responsible for the bitter tang and high toxicity, Lima et al. [1] showed some toxicological effects of *S. brasiliensis* only at very high doses (i.e., 2 g/kg). In a recent study, Dos Santos et al. [3] performed a regional ethnopharmacological use of the infusion of *S. brasiliensis* stems in Mato Grosso (Brazil) and demonstrated that the crude hydroethanol extract reduced the hyperglycemia and glycosuria in diabetic mice. On the other hand, our continuous search for evidences for the traditional use of Brazilian species led us to hear about the popular use of *S. brasiliensis* to treating pain as a result of toothache. On that basis, the aim of the present work was to investigate the antinociceptive effect of *S. brasiliensis* extract and its previously isolated saponins: Cayaponoside A1, cayaponoside B4, cayaponoside D, and siolmatroside I, and suggest the mechanism of their antinociceptive activity. In this regard we used atropine (cholinergic antagonist), naloxone (opioid antagonist), or L-NAME (nitric oxide synthase inhibitor) to evaluate the participation of these pathways in the antinociceptive effect of *S. brasiliensis*. It is important to mention that siolmatroside I (a dammarane type saponin) was described by our research group for the first time in the plant kingdom.

## 2. Results

### 2.1. Assessment of Side Effects and Toxicity

A single oral administration of HE, EtOAc, or the isolated saponins (SI, D, B4, and A1) were evaluated against a possible toxic effect when administered orally. Doses of 150 mg/kg of extracts or 10 mg/kg of each isolated saponins were used. After periods of 1, 3, 6, and every 24 h until five days post-oral administration animals were evaluated regarding behaviour alterations. We also evaluated food and water intake every 24 h until the fifth day. Results obtained indicated that none of the extract or isolated saponins induced compartmental alterations, did not change the amount of food and water intake, and did not induce any mucosal lesion five days after their administration, presenting visual conditions similar to those for the vehicle (supplementary material, Tables S1–S4).

### 2.2. Antinociceptive Effect of HE, EtOAc, SI, D, B4, and A1 in the Hot Plate Test

**Figure 1:**
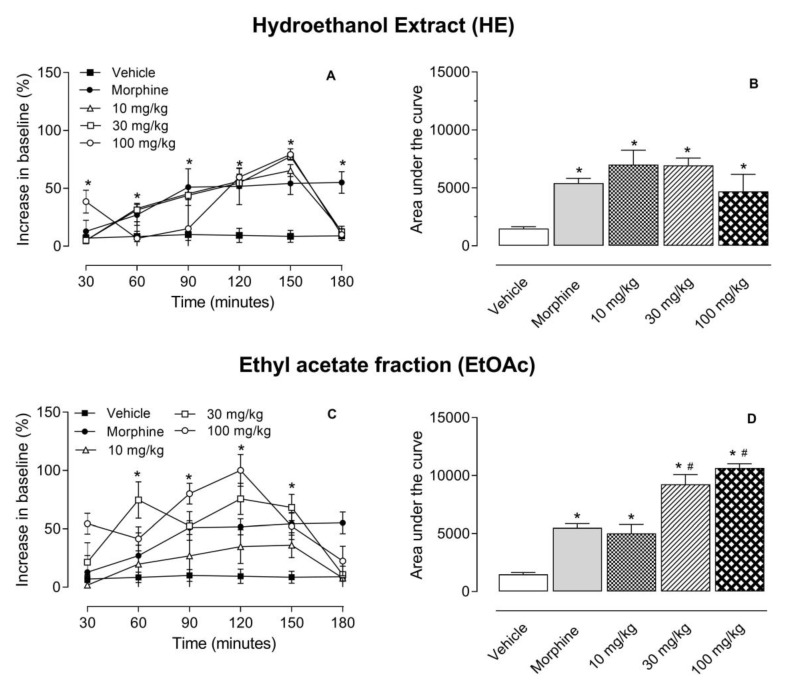
Effects of hydroethanol extract (HE, graphs **A** and **B**) or ethyl acetate fraction (EtOAc, graphs **C** and **D**) of *S. brasiliensis* in the hot plate model. Animals were orally pretreated with different doses of HE, EtOAc, morphine (2.5 mg/kg) or vehicle. The results are presented as mean ± SD. (*n* = 6 per group) of increase in baseline (graphs **A** and **C**) or area under the curve (graphs **B** and **D**) calculated by Prism Software 5.0. Statistical significance was calculated by ANOVA followed by Dunnett’s test. * *p* < 0.05 when comparing to vehicle-treated group; # *p* < 0.05 when comparing treated mice with the morphine-treated group.

The hot plate model was used to evaluate the supraspinal antinociceptive effect of the tested substances. As it can be observed in Figure 1A and B, all three doses of HE and EtOAc significantly increased the time necessary for mice to respond to the stimulus. Maximal effects were observed at 150- or 120-min post-administration, respectively, and they returned to basal levels after this period. When data obtained in time course assays were converted to a graph of area under the curve (AUC) it could be noted that all doses increased these values. Furthermore, higher doses (30 and 100 mg/kg) of EtOAc were significantly even when compared to the morphine-treated group (Figure 1C and D). Although a lower dose (30 mg/kg) of HE presented higher area under the curve values than higher dose (100 mg/kg) (7001 ± 1260 versus 4697 ± 1483) a statistical significance between both groups was not observed (Figure 1A and B).

**Figure 2:**
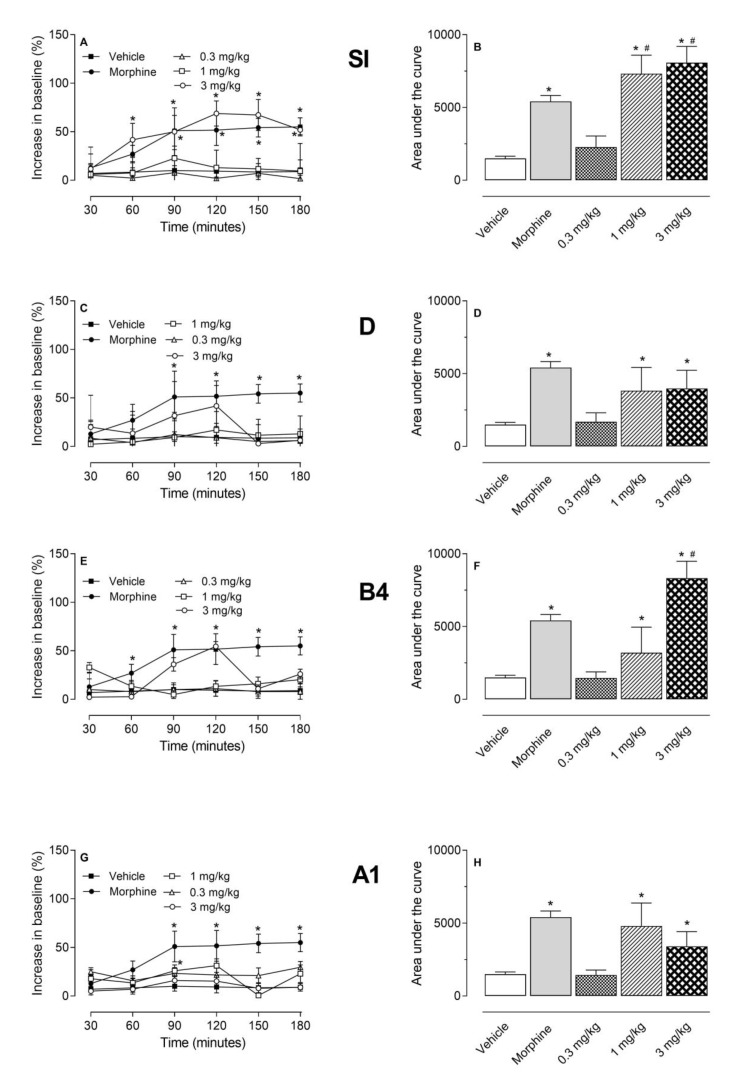
Effects of Saponins isolated from the ethyl acetate fraction of the stems of *S. brasiliensis*: siolmatroside I (SI), cayaponoside D (D), cayaponoside B4 (B4), and cayaponoside A1 (A1) in the hot plate model. Animals were orally pretreated with different doses of A1, B4, D, I, morphine (2.5 mg/kg), or vehicle. The results are presented as mean ± SD. (*n* = 6 per group) of increase in baseline (graphs **A**, **C**, **E,** and **G**) or area under the curve (graphs **B**, **D**, **F,** and **H**) calculated by Prism Software 5.0. Statistical significance was calculated by ANOVA followed by Dunnett´s test. * *p* < 0.05 when comparing to vehicle-treated group; # *p* < 0.05 when comparing treated mice with the morphine-treated group.

The next step was the evaluation of the saponins isolated from ethyl acetate fraction using this same model. The doses were chosen based on the yield of each saponin after isolation from the ethyl acetate fraction. Data shown in Figure 2 demonstrated that doses of 1 and 3 mg/kg of all saponins presented a significant antinociceptive effect increasing the AUC. It is interesting to note that SI (at the doses of 1 and 3 mg/kg) presented an effect higher than that observed for the positive control group (morphine-treated mice).

### 2.3. Investigation of the Mechanism of Action of EtOAc, SI, D, B4, and A1 in the Hot Plate Model

**Figure 3:**
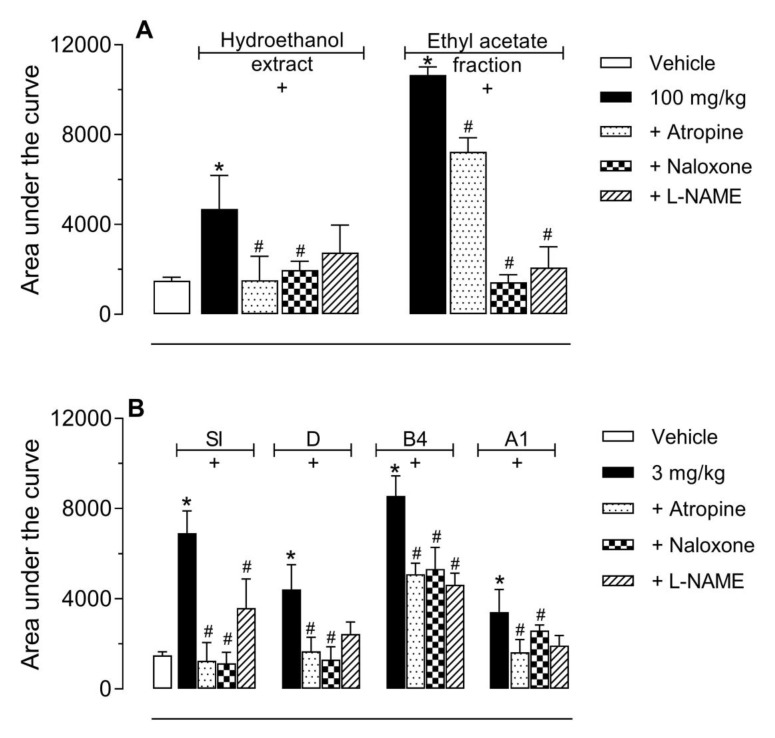
Effects of different antagonists on the antinociceptive activity of HE, EtOAc (graph **A**), siolmatroside I (SI), cayaponoside D (D), cayaponoside B4 (B4), and cayaponoside A1 (A1) (graph **B**) in the hot plate model. Animals were pretreated with atropine (1 mg/kg, i.p.), naloxone (1 mg/kg, i.p.) or L-NAME (3 mg/kg, i.p.), 15 min prior to oral administration of HE, EtOAc (100 mg/kg), SI, D, B4, and A1 (3 mg/kg). The results are present as mean ± SD. (*n* = 6 per group) of the area under the curve calculated by Prism Software 5.0. Statistical significance was calculated by ANOVA followed by Dunnett´s test. * *p* < 0.05 when comparing HE, EtOAc, SI, D, B4, and A1-treated mice to the vehicle-treated group; # *p* < 0.05 when comparing antagonist or inhibitor pretreated mice with the HE, EtOAc, SI, D, B4, or A1-treated group.

As the ethanol extract, ethyl acetate fraction and its isolated saponins (SI, D, B4, and A1) showed that the significant antinociceptive effect was decided to further investigate the role of different nociceptive pathways involved in the transmission of nociceptive stimulus or the activation of pathways involved in the control of nociception. None of the receptor antagonists (atropine and naloxone) or enzyme inhibitor (L-NAME) demonstrated any antinociceptive effect per se in the hot plate model (Data not shown). As the intention was to observe an inhibitory effect, we decided to use the higher dose of the extract, fraction (100 mg/kg), or isolated saponins (3 mg/kg). The pretreatment with atropine (muscarinic receptor antagonist, 1 mg/kg, i.p.) or naloxone (opioid receptor antagonist, 1 mg/kg, i.p.) reversed the antinociceptive effect of HE and EtOAc (Figure 3A), SI, D, B4, and A1 (Figure 3B). The inhibitor of nitric oxide synthase enzyme (L-NAME, 3 mg/kg, i.p.) reversed the antinociceptive effect EtOAc (Figure 3A), SI, B4, and A1 (Figure 3B).

### 2.4. Antinociceptive Effect of HE, EtOAc, SI, D, B4, and A1 in the Capsaicin- or Glutamate-Induced Nociception

**Figure 4:**
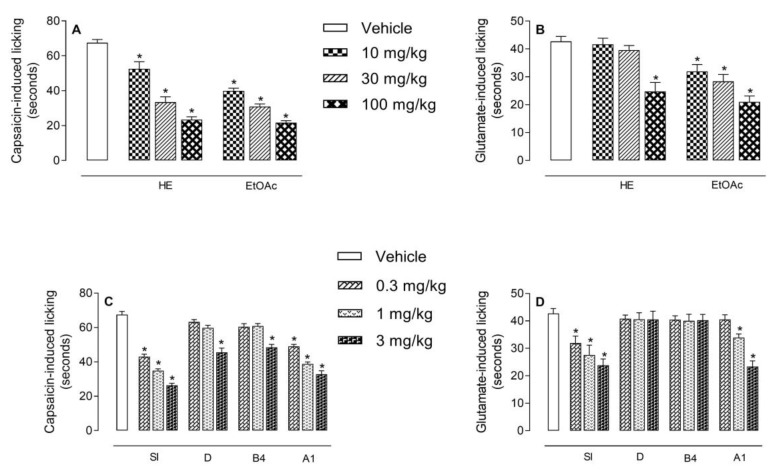
Antinociceptive effect of HE, EtOAc (graphs **A** and **B**), siolmatroside I (SI), cayaponoside D (D), cayaponoside B4 (B4), and cayaponoside A1 (A1) (graphs **C** and **D**) on the licking response induced by capsaicin or glutamate in mice, respectively. Animals were pretreated with different doses of HE, EtOAc, SI, D, B4, and A1 or vehicle 60 min before the injection of capsaicin (1.6 ng/paw) or glutamate (3.7 ng/paw). The results are presented as mean ± SD. (*n* = 6 per group) of the time that the animal spent licking the capsaicin-injected paw. Statistical significance was calculated by ANOVA followed by Dunnett´s test. * *p* < 0.05 when compared to vehicle-treated mice.

The results depicted in Figure 4A show that HE and EtOAc produced a significantly and dose-dependent reduction in the capsaicin-induced neurogenic pain. Pretreatment of animals with HE (at 10, 30, or 100 mg/kg doses) significantly reduced the paw licking induced by capsaicin by 22.2%, 50.4%, and 65.3% (52.1 ± 4.1; 33.5 ± 3.1, and 23.4 ± 1.7 s, respectively). Same doses of EtOAc demonstrated 40.7%; 54.2% and 67.8% reduction (40 ± 1.5; 30.9 ± 1.5; 21.7 ± 1.2 s, respectively) when compared to vehicle-treated animals (67.5 ± 1.9 s). Oral treatment of mice with A1 or SI significantly inhibited the capsaicin-induced licking pain at the doses of 0.3, 1, and 3 mg/kg, with the following results: 43.1 ± 1.4 (36.1%), 34.9 ± 1.1 (48.3%), 26.4 ± 1.2 (60.9%), and 49.0 ± 1.2 (27.4%), 38.8 ± 1.1 (42.5%), 32.8 ± 2.0 s (51.4%), respectively, when comparing with vehicle-treated mice (67.5 ± 1.9 s). Only the dose of 3 mg/kg of B4 and D demonstrated a significant effect (Figure 4C).

We also observed that HE (at 100 mg/kg, p.o.) produced a significant reduction (41.9%) of glutamate-induced licking response (24.8 ± 3.2 s) when compared to vehicle-treated mice (42.7 ± 1.8 s). While EtOAc, at all doses, significantly and dose-dependently inhibited the glutamate-induced pain behavior (31.9 ± 2.5, 28.4 ± 2.9, and 21 ± 2.1 s, to 10, 30, and 100 mg/kg, respectively) when comparing with the vehicle-treated group, leading to an inhibition of 25.3%, 33.5%, and 50.8%, respectively (Figure 4B). Among the isolated saponins, only SI (0.3, 1, and 3 mg/kg p.o.) was effective in reducing (31.9 ± 2.6, 27.6 ± 3.6, and 23.8 ± 5 s) the glutamate-induced nociceptive response corresponding to an inhibition of 5.3%, 34.6%, and 44.3%, respectively. The cayaponoside A1 reduced the paw licking at the doses of 1 and 3 mg/kg (34.0 ± 1.5 and 23.3 ± 2.1 s) producing a reduction of 20.4% and 45.4%, respectively (Figure 4D).

## 3. Discussion

This study investigated the antinociceptive activity of *S. brasiliensis* confirming its popular use and contributing to the pharmacological knowledge of this plant.

To the best of our knowledge this is the first study showing that hydroethanol extract (HE) of *S. brasiliensis* stems, one of its fraction (EtOAc) and two saponins isolated from this fraction (A1 and SI) have antinociceptive activity when administered orally in different models of thermal and chemical nociception in mice.

When a chemical, mechanical, or thermal stimulus occurs in mice paw, there is activation of nociceptors that transmit nociceptive information to the somatosensory cortex, for example, present in the central nervous system (CNS) producing an organized response, resulting in an elevation of motor response and/or licking of the paw [4]. The administration of HE, EtOAc, SI, D, B4, and A1, produced a rapid effect and time course of action similar to morphine.

Several saponins have already been related to the antinociceptive/anti-inflammatory activity. Examples to cite are saponins from *Ipomoea involucrate* [5], *Pterodone marnatus* [6], and Xeromphisnilotica [7]. Saponins isolated from *S. brasiliensis* in this study belong to the cucurbitane (A1, B4, D) and dammarane (SI) types. Cucurbitanes have been shown to possess a good antinociceptive and anti-inflammatory action as demonstrated recently in a *Momordica charantia* review paper [8].

Knowing that several endogenous systems are involved in pain control, the mechanism of antinociceptive action of HE, EtOAc, SI, D, B4, and A1 was also investigated. Atropine, a non-selective muscarinic receptor antagonist, was used to assess the involvement of the cholinergic system. Some studies show that activation of the muscarinic receptor (subtype M2) may reduce the response of peripheral nociceptors in front of noxious stimuli. The stimulation of the M2 and M4 muscarinic receptors in the dorsal horn of the spinal cord contributes to the analgesic effect through the release of inhibitory interneurons, reducing nociceptive transmission [9]. Our results showed the reversal of the antinociceptive effect of HE, EtOAc, SI, D, B4, and A1 when atropine was used suggesting that the effects of extract, fraction, or isolated saponins may involve, at least in part, activation of muscarinic receptors and consequently cholinergic pathway.

Our data also indicated that the opioid system could be involved in the antinociception caused by HE, EtOAc, SI, D, and B4. Opioid receptors (μ, δ, and κ) can be found in peripheral, spinal, and supraspinal regions. The downward pain modulation also involves the participation of opioid receptors, which promotes reduction in synaptic release of γ-aminobutyric acid (GABA) to the spinal cord rostral medial projections and periaqueductal gray; subsequently leading to spinal disinhibited projections of adrenergic neurons in the locus coeruleus [10]. In this context, the extract and isolated substances tested in this work may exert their antinociceptive effects through the opioid system at the peripheral level, spinal, and/or supraspinal. Literature reviews have already demonstrated that saponins may present an antinociceptive activity. The investigation of the mechanism of action of a *Polygonum verticillatum* methanol extract rich in saponins showed the involvement of the opioid pathway [11], similarly to what was observed in our study for the ethanol extract of *S. brasiliensis*, its EtOAc fraction, two curcubitane, and one dammarane type of saponin.

The knowledge of the dual role of nitric oxide (NO) in nociceptive transmission and its contribution to antinociception by opioid pain killers and anti-inflammatory drugs motivated us to assess whether the antinociception of HE, EtOAc, SI, D, B4, and A1 involve nitrergic pathway. NO present in the dorsal horn of the spinal cord can stimulate the release of neurokinin A, substance P, CGRP, and glutamate by primary afferent fibers facilitating nociceptive transmission. Activation of NMDA type receptors by NO is involved in central sensitization mechanism [10]. In this context, our results showed that the antinociceptive effect of EtOAc, SI, B4, and A1 was reversed by pretreatment with L-NAME suggesting that the antinociceptive effect of EtOAc and the three substances involves the inhibition of NOS activity and/or indirect reduction in NO levels.

Hydroethanol extract, ethyl acetate fraction, and the isolated substances SI and A1 also presented an inhibitory effect against the neurogenic nociception induced by intraplantar injection of capsaicin. It is an amine extracted from red pepper that stimulates nerve endings causing intense thermal and nociceptive pain [9,11,12]. Studies have shown that capsaicin acts through vanilloid receptor type-1 (TRPV-1) expressed on nociceptive fibers [13] and in the dorsal root ganglion of the spinal cord, trigeminal ganglia, and CNS [14]. The stimulation of TRPV-1 receptors is mediated by the release of several neurotransmitters, including glutamate and substance P, from the peripheral and central terminals of primary sensory neurons thus contributing to nociceptive processing [15,16]. Our data suggest that extracts and isolated saponins reduce licking behavior induced by capsaicin. We have no means to attest that the observed effect is mediated through a direct action on peripherals and/or central vanilloid receptors, but we can suggest that part of the effect is mediated by capsaicin pathway.

We also suggest that at least part of the inhibitory effect observed can also occur due to an effect against the glutamate pathway since it was observed that EtOAc reduced glutamate-induced licking response. It is most probably that the effect of *S. brasiliensis* extract, fraction, and SI occurs due to a direct combination of effects in the capsaicin and glutamate systems. It is also suggested that isolated saponins D and B4 do not present an effect against both neurogenic substances.

Taken together our data suggest *S. brasiliensis* and the isolated substances siolmatroside I (SI), cayaponoside D (D), cayaponoside B4 (B4), and cayaponoside A1 (A1) present significant antinociceptive effects. Cholinergic and opioidergic pathways seem to be involved in their mechanism of action. We also demonstrated that the siolmatroside I was the most active saponin identified in the plant. Taken together our data corroborate the traditional use of the plant and expands the information regarding its use.

## 4. Methods

### 4.1. Plant Material

Stems of *S. brasiliensis* were collected in Jangada/Mato Grosso State, Brazil and identified by Dr. Vali Joana Pott (Department of Biology, UFMT, Cuiabá, Brazil). A voucher specimen was deposited at the herbarium of the UFMT under the number CGMS: 31643.

### 4.2. Extraction and Isolation

**Figure 5:**
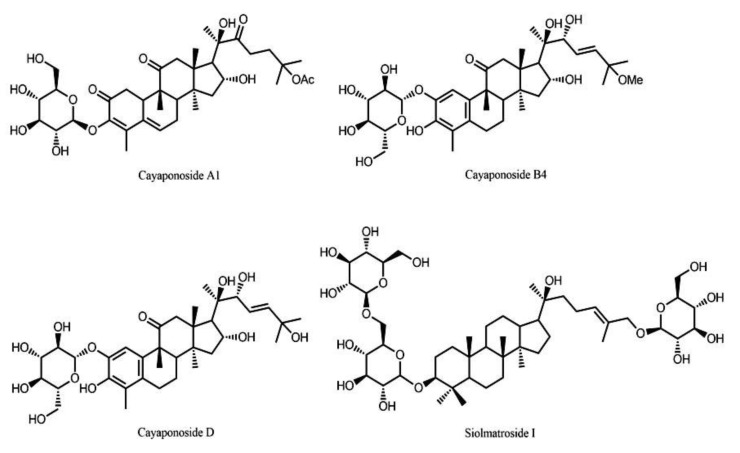
Saponins isolated from the ethyl acetate fraction of the stems of *S. brasiliensis*: Cayaponoside A1, cayaponoside B4, cayaponoside D, and siolmatroside I.

Air dried stems of *S. brasiliensis* (2.07 kg) were powdered and subjected to extraction with a mixture of EtOHcH_2_O (7:3) (3 × 6 L) (named hydroethanol, HE extract). The HE extract was concentrated under reduced pressure and circulation oven (45 °C). HE (149 g) was suspended in a mixture of MeOH:H_2_O (1:1) (1 L) and subjected to a liquid–liquid partition between chloroform, ethyl acetate, and n-butanol. Cayaponoside A1 [17] (A1), cayaponoside B4 [18] (B4), cayaponoside D [17] (D), and siolmatroside I (SI) were isolated by us from the ethyl acetate fraction, as described previously [3] (Figure 5).

### 4.3. Animals

Animals used in this study (Swiss Webster mice (25–30g)) were donated by Instituto Vital Brazil (Niterói, Rio de Janeiro, Brazil) and maintained in a room with light-dark cycle of 12 h, 22 ± 2 °C, 60% to 80% humidity and food and water provided ad libitum. Before each test, the animals were acclimatized to the laboratory for at least 1 h. Twelve hours before each experiment the animals received only water in order to avoid food interference with substances absorption. Animals were daily monitored to assess their physical conditions and that with any signs of suffering were euthanized. None of the animals used became severely ill or died at any time prior to the experimental endpoint. At the end of each assay, animals were euthanized following AVMA guidelines. The experimental protocols used in this work have been carried out in accordance with the Guide for the Care and Use of Laboratory Animals as adopted and promulgated by the US National Institutes of Health, and were approved by the rules advocated by Law 11,794, of 8 October 2008 by the National Council of Animal Experimentation Control (CONCEA) and were approved by the Ethics Committee of Animal Use (CEUA)/UFRJ and received the number 31/19 and 34/19.

### 4.4. Extracts, Isolated Substances, and Control Drugs’ Administration

Hydroethanol extract, fractions, and the isolated substances were dissolved in dimethylsulfoxide (DMSO, Sigma-Aldrich, St. Louis, MO, USA) in order to prepare a stock solution at 200 mg/mL. The hydroethanol extract (HE) and the ethyl acetate fraction (EtOAc) were tested at doses of 10, 30, or 100 mg/kg. Siolmatroside I (SI), cayaponoside D (D), cayaponoside B4 (B4), and cayaponoside A1 (A1) were tested at doses of 0.3, 1, or 3 mg/kg. All of them were administered by oral gavage in a final volume of 0.1 mL per animal randomly divided into groups of 6 to 8 mice. The final amount of DMSO used in higher dose (100 mg/kg) had no effect per se. Morphine (2.5 mg/kg) was used as a reference drug and was administered by oral gavage at the intervals indicated in each protocol. Control group was the given vehicle (ultrapure water with the same amount of DMSO used in higher dose).

### 4.5. Drugs and Reagents

Morphine sulfate and naloxone hydrochloride (both dissolved in phosphate buffer saline, PBS) were kindly provided by Cristália (São Paulo, Brazil). Atropine sulfate monohydrate (in PBS), capsaicin (in DMSO), l-glutamic acid hydrochloride (glutamate, in PBS), l-nitroarginine methyl ester (L-NAME, in PBS) were purchased from Sigma-Aldrich (St. Louis, MO, USA). All drugs were diluted just before their use.

### 4.6. Acute Toxicity

Acute toxicity parameters were determined following the method described by Lorke [19]. Oral dose of the HE, EtOAc (150 mg/kg), or isolated saponins (SI, D, B4, A1, 10 mg/kg) were administered to groups of ten mice (five males and five females). Parameters such as convulsion, sedation, reflex, hyperactivity, increased or decreased respiration, and food and water intake were observed at 1, 3, 6, 12, and every 24 h over a period of five days to analyze the behaviour of the animals. After that, mice’s stomachs were removed in order to search for ulcers (single or multiple erosion, ulcer or perforation) and instances of hyperemia were counted.

### 4.7. Hot Plate Test

Mice were orally treated with substances, vehicle, or morphine and immediately placed on a hot plate apparatus (Insight Equipment, Brazil) set at 55 ± 1 °C. The time necessary for mice that licked their fore- and/or hind-paws (named reaction time) was measured at intervals of 30 min post-oral administration. Baseline was calculated with the use of measurements obtained at 60 and 30 min before the administration of the compounds, vehicle, or morphine and considered as a normal reaction of the animal to the temperature. The calculation of area under the curve (AUC) of responses from 30 min after drug administration until the end of the experiment was used to indicate the antinociception. The following formula based on the trapezoid rule was used to calculate the AUC: AUC = 30 × IB ((min 30) + (min 60) +…+ (min 180)/2), where IB is the increase from baseline (in %) [20,21].

### 4.8. Analysis of the Mechanisms of Action of Hydroethanol Extract (HE), Ethyl Acetate Fraction (EtOAc), and its Isolated Saponins

To investigate the involvement of the cholinergic, opioid, or nitrergic system in the mechanism of action of substances, some specific receptor antagonists and/or enzyme inhibitors were used. After 15 min of intraperitoneal administration of atropine (muscarinic receptor antagonist, 1 mg/kg), naloxone (opioid receptor antagonist, 1 mg/kg), or l-nitro arginine methyl ester (L-NAME, inhibitor of nitric oxide synthase enzyme, 3 mg/kg) mice received oral administration of HE or EtOAc (100 mg/kg each), SI, D, B4, or A1 (3 mg/kg each). Based on data from literature [22,23] and previous data from our own laboratory [24], dose response curves with agonists and respective antagonist were previously constructed and the dose of antagonist that reduced in 50% the agonist effect was chosen for these assays. The antinociceptive effect was evaluated in the hot plate test as described above.

### 4.9. Capsaicin- and Glutamate-Induced Nociception

The animals were pretreated 60 min before the intraplantar injection of capsaicin or glutamate with substances or vehicle by oral gavage. After treatment, mice received an intraplantar injection of capsaicin (20 μL, 1.6 μg/paw) or glutamate (20 μL, 3.7 ng/paw). Immediately animals were individually placed in a transparent box and the time that the animal kept licking the capsaicin- or glutamate-injected paw was recorded during a period of 5 min [25] and 15 min [26], respectively. The time mice spent licking the injected paw was recorded and this was considered as the nociceptive reaction.

### 4.10. Statistical Analysis

Experimental groups were composed of 6–8 mice and the results were presented as mean ± S.D. Statistical significance between groups was determined using the application of analyses of variance (ANOVA) followed by Dunnett´s post-test. Differences were considered significant (*) when *p* < 0.05. The area under the curve (AUC) was calculated using Prism Software 5.0 (Graph Pad Software, La Jolla, CA, USA).

## 5. Conclusion

Our work validated for the first time the antinociceptive activity in peripheral and central models of analgesia of *S. brasiliensis* ethanol extract, its ethyl acetate fraction, and its isolated saponins cayaponoside A1, cayaponoside B4, cayaponoside D, and siolmatroside I. The actions are mediated by opioid, nitrergic, and cholinergic systems and, in part by vanilloid receptors. Our study confirms that this species may be used for treating pain processes corroborating with its traditional use and contributing to the pharmacological knowledge of this Brazilian species.

## Supplementary Materials

The following are available online. Table S1: Toxic effects of hydroethanol extract (HE), ethyl acetate fraction (EtOAc), siolmatroside I (SI), cayaponoside D (D), cayaponoside B4 (B4) and cayaponoside A1 (A1) observed at 1, 3, 6 or 12 hours post-oral administration. Table S2: Toxic effects of hydroethanol extract (HE), ethyl acetate fraction (EtOAc), siolmatroside I (SI), cayaponoside D (D), cayaponoside B4 (B4) and cayaponoside A1 (A1) observed at 1, 2, 3, 4 or 5 days post-oral administration; Table S3: Effects of hydroethanol extract (HE), ethyl acetate fraction (EtOAc), siolmatroside I (SI), cayaponoside D (D), cayaponoside B4 (B4) and cayaponoside A1 (A1) in water and food intake observed at 1, 2, 3, 4 or 5 days post-oral administration; table S4: Effects of hydroethanol extract (HE), ethyl acetate fraction (EtOAc), siolmatroside I (SI), cayaponoside D (D), cayaponoside B4 (B4) and cayaponoside A1 (A1) in the presence of ulcers or hyperemia 5 days post-oral administration.
