# Supplementary material for: Study on the Antinociceptive Activity and Mechanism of Action of Isolated Saponins from Siolmatra brasiliensis (Cogn.) Baill
Source: Molecules. 2019 Dec 14;24(24):4584. doi: 10.3390/molecules24244584 (PMC6943633; doi:10.3390/molecules24244584)
Supplement: Supplementary file 1 [file molecules-24-04584-s001.pdf]

Supplementary Materials

Study on the Antinociceptive Activity and Mechanism of Action of Isolated Saponins from *Siolmatra brasiliensis* (Cogn.) Baill

Thais Biondino Sardella Giorno [1,2], Carlos Henrique Corrêa dos Santos [3], Mario Geraldo de Carvalho [3], Virgínia Cláudia da Silva [4], Paulo Teixeira de Sousa Jr. [4], Patricia Dias Fernandes [1,2,*] and Fabio Boylan [5]

1 Federal University of Rio de Janeiro, Institute of Biomedical Sciences, Laboratory of Pharmacology of Pain and Inflammation, Rio de Janeiro 21941-902, Brazil; thais.sardella.farma@hotmail.com (T.B.S.G); patricia.dias@icb.ufrj.br (P.D.F.)

2 Federal University of Rio de Janeiro, Institute of Biomedical Sciences, Graduate Program in Pharmacology and Medicinal Chemistry, Rio de Janeiro 21941-902, Brazil; thais.sardella.farma@hotmail.com (T.B.S.G); patricia.dias@icb.ufrj.br (P.D.F.)

3 Federal Rural University of Rio de Janeiro, Department of Chemistry, Seropédica, 23890-000, Brazil; caio.chcs@msn.com (C.H.C.d.S.); mgeraldo@ufrrj.br (M.G.d.C.)

4 Federal University of Mato Grosso, Department of Chemistry, Cuiabá, 78935-901, Brazil; vcsvirginia@yahoo.com.br (V.C.d.S.); pauloteixeiradesousa@gmail.com (P.D.T.d.S.J.)

5 Trinity College Dublin, Trinity Biomedical Sciences Institute, School of Pharmacy and Pharmaceutical Sciences, Dublin 2, Ireland; fabio.boylan@tcd.ie (F.B.)

* Correspondence: patricia.dias@icb.ufrj.br; Tel.: +5521-3938-6442

Table 1. Toxic effects of hydroethanol extract (HE), ethyl acetate fraction (EtOAc), siolmatroside I (SI), cayaponoside D (D), cayaponoside B4 (B4) and cayaponoside A1 (A1) observed at 1, 3, 6 or 12 hours post-oral administration.

**Substance/**	**Convulsion**				**Sedation**				**Reflex**				**Hyperactivity**				**Respiration**			
**effect/hour**	**1**	**3**	**6**	**12**	**1**	**3**	**6**	**12**	**1**	**3**	**6**	**12**	**1**	**3**	**6**	**12**	**1**	**3**	**6**	**12**
HE	0	0	0	0	0	0	0	0	0	0	0	0	0	0	0	0	0	0	0	0
EtOAc	0	0	0	0	0	0	0	0	0	0	0	0	0	0	0	0	0	0	0	0
SI	0	0	0	0	0	0	0	0	0	0	0	0	0	0	0	0	0	0	0	0
D	0	0	0	0	0	0	0	0	0	0	0	0	0	0	0	0	0	0	0	0

B4	0	0	0	0	0	0	0	0	0	0	0	0	0	0	0	0	0	0	0	0
A1	0	0	0	0	0	0	0	0	0	0	0	0	0	0	0	0	0	0	0	0

Data represents the number of mice with the respective effect in a total of 10 animals.

Table 2. Toxic effects of hydroethanol extract (HE), ethyl acetate fraction (EtOAc), siolmatroside I (SI), cayaponoside D (D), cayaponoside B4 (B4) and cayaponoside A1 (A1) observed at 1, 2, 3, 4 or 5 days post-oral administration.

**Substance/**	**Convulsion**					**Sedation**					**Reflex**					**Hyperactivity**					**Respiration**				
**effect/hour**	**1**	**2**	**3**	**4**	**5**	**1**	**2**	**3**	**4**	**5**	**1**	**2**	**3**	**4**	**5**	**1**	**2**	**3**	**4**	**5**	**1**	**2**	**3**	**4**	**5**
HE	0	0	0	0	0	0	0	0	0	0	0	0	0	0	0	0	0	0	0	0	0	0	0	0	0
EtOAc	0	0	0	0	0	0	0	0	0	0	0	0	0	0	0	0	0	0	0	0	0	0	0	0	0
SI	0	0	0	0	0	0	0	0	0	0	0	0	0	0	0	0	0	0	0	0	0	0	0	0	0
D	0	0	0	0	0	0	0	0	0	0	0	0	0	0	0	0	0	0	0	0	0	0	0	0	0
B4	0	0	0	0	0	0	0	0	0	0	0	0	0	0	0	0	0	0	0	0	0	0	0	0	0
A1	0	0	0	0	0	0	0	0	0	0	0	0	0	0	0	0	0	0	0	0	0	0	0	0	0

Data represents the number of mice with the respective effect in a total of 10 animals.

Table 3. Effects of hydroethanol extract (HE), ethyl acetate fraction (EtOAc), siolmatroside I (SI), cayaponoside D (D), cayaponoside B4 (B4) and cayaponoside A1 (A1) in water and food intake observed at 1, 2, 3, 4 or 5 days post-oral administration.

**Substance/**	**Food (in grams)**					**Water (in ml)**				
**day**	**1**	**2**	**3**	**4**	**5**	**1**	**2**	**3**	**4**	**5**
Vehicle	9.9 ± 1.4	7.4 ± 0.5	11.8 ± 1.8	10.5 ± 2.8	8.9 ± 0.8	58.1 ± 6.9	64.1 ± 9.9	64.3 ± 10.2	55.5 ± 7.8	66.4 ± 7.1
HE	9.8 ± 1.1	12.4 ± 3.1	9.8 ± 1.1	11.9 ± 0.5	12.8 ± 1.9	61.3 ± 7.7	66.4 ± 3.5	55.3 ± 6.8	55.1 ± 4.4	58 3 ± 9.9
EtOAc	7.3 ± 2.8	8.1 ± 3.1	8.8 ± 3.3	8.7 ± 2.1	9.9 ± 1.1	55.8 ± 8.7	71.1 ± 8.9	75.8 ± 12.4	66.9 ± 9.3	77.3 ± 9.2
SI	6.9 ± 3.1	7.3 ± 3.1	11.7 ± 4.0	9.9 ± 1.7	8.2 ± 3.3	59.4 ± 6.2	82.4 ± 11.2	78.4 ± 9.3	88.8 ± 11.5	73.5 ± 9.9
D	8.5 ± 3.0	8.9 ± 1.8	7.8 ± 1.4	11.7 ± 3.8	8.9 ± 1.0	62.7 ± 3.8	81.7 ± 11.1	77.9 ± 12.5	81.3 ± 13.5	77.3 ± 11.9
B4	10.1 ± 0.9	9.5 ± 2.1	5.9 ± 2.8	12.9 ± 3.1	9.3 ± 3.4	66.9 ± 7.3	81.6 ± 14.8	77.9 ± 12.6	71.6 ± 13.4	79.3 ± 9.6
A1	8.7 ± 1.9	11.4 ± 3.8	8.8 ± 1.9	14.9 ± 3.1	11.8 ± 2.7	72.4 ± 9.9	77.1 ± 9.3	81.1 ± 8.3	71.5 ± 9.3	84.7 ± 9.9

Data represents media ± SD (n=10) of amount of food (in mg) or water (in ml) intake every day.

Table 4. Effects of hydroethanol extract (HE), ethyl acetate fraction (EtOAc), siolmatroside I (SI), cayaponoside D (D), cayaponoside B4 (B4) and cayaponoside A1 (A1) in the presence of ulcers or hyperemia 5 days post-oral administration.

**Substance**	**Ulcer**	**Hyperemia**
Vehicle	0	0
HE	0	0
EtOAc	0	0
SI	0	0
D	0	0
B4	0	0
A1	0	0

Data represents of number of ulcers or hyperemia per animal.
